# Adult Renal Transplantation in a Patient 28 Years after Heart Transplantation as a Neonate for Hypoplastic Left Heart Syndrome

**DOI:** 10.1155/2022/7532199

**Published:** 2022-04-05

**Authors:** Joseph Licata, Shane Michael Roberts, Piotr Janicki, Dmitri Bezinover

**Affiliations:** Division of Anesthesiology and Perioperative Medicine, Resident PGY-4 Penn State Milton S. Hershey Medical Center, 500 University Dr., Hershey, PA 17033, USA

## Abstract

We present a case of kidney transplantation in a 28-year-old patient who received a heart transplant at 7 weeks of age due to hypoplastic left heart syndrome. The patient's renal insufficiency was the result of chronic immunosuppression and hypertension. The almost 28-year-old graft demonstrated very good function. This patient represents as one of the longest pediatric cardiac graft recipients living without any significant functional limitations.

## 1. Introduction

Hypoplastic left heart syndrome (HLHS) is a rare congenital cardiac defect characterized by hypoplasia of the left ventricular outflow tract usually accompanied by hypoplasia or atresia of the mitral and/or aortic valves. Untreated, this condition is incapable of supporting systemic circulation [1]. The syndrome, initially described by Lev in 1952 [2], has an incidence of between 0.016 and 0.036% [3, 4]. Historically, heart transplantation (HT) was the only viable treatment for these patients. Contemporary management of HLHS involves a staged palliative repair performed in the neonatal period and early childhood.

HT performed in infancy is generally associated with good survival and an acceptable quality of life although long-term immunosuppression can lead to significant complications including chronic kidney disease (CKD). CKD is not uncommon after HT in both adults and children, affecting more than half of these patients [5]. In children, the overall prevalence of end stage kidney disease (ESKD) requiring dialysis and/or kidney transplantation (KT) after HT has been reported to be as much as 4% [6–8]. The risk for developing ESKD after HT in children has been reported to be 3% at 10 years and 16% after 20 years [8]. We describe a case of KT in a patient 28 years after HT for HLHS. This is one of the longest living HT recipients surviving without the need for retransplantation.

## 2. Case Description

A 29-year-old 78 kg female presented for a living donor kidney transplant (LDKT). She was born with HLHS and underwent a HT at 7 weeks of age. Posttransplant immunosuppressive therapy included tacrolimus and azathioprine since transplantation. Her ESKD was presumably caused by tacrolimus toxicity. Other medical comorbidities included hypertension (a contributing factor for ESKD) managed with amlodipine and familial hypocalcemia. A pacemaker was inserted at 18 years of age due to bradycardia. The patient's renal function rapidly deteriorated 4 months prior to KT requiring dialysis.

Six months prior to KT, a transthoracic echocardiogram demonstrated normal cardiac function (EF of 60-65%) with mild aortic and mild to moderate mitral regurgitation. Preoperatively, she displayed very good physical exercise capacity. She was able to climb a flight of stairs without getting short of breath (4-6 METs).

On the day of her surgery, immunosuppression was initiated with 1000 mg methylprednisone infused over 60 minutes.

Anesthesia was performed using institutional protocol. Propofol 2 mg/kg, fentanyl 2 mcg/kg, and cis-atracurium 0.15 mg/kg were used for anesthesia induction. After tracheal intubation, mechanical ventilation (with 35–40% oxygen/air mixture) was started. Anesthesia was maintained with isoflurane and fentanyl boluses. Additional cis-atracurium (0.05–0.1 mg/kg) was used as necessary.

After intubation, a left radial arterial line and a left internal jugular 7-French triple lumen catheter (CVC) were placed without complications. Additionally, thymoglobulin (150 mg) was infused through the CVC over 6 hours.

An intraoperative transesophageal echo (TEE) demonstrated a mildly dilated right ventricle (RV) with preserved systolic function and an ejection fraction of 60-65%. Both atria were moderately dilated, and there was mild to moderate mitral regurgitation (Video 1), mild aortic (Video 2) and pulmonic regurgitation, and moderate tricuspid regurgitation (Video 3) with mild pulmonary hypertension (pulmonary artery systolic pressure was 38.3 mmHg).

The surgical procedure was uneventful. Central venous pressure remained within a range of 12-15 cm H_2_O. TEE was used to monitor cardiac function throughout the case and did not demonstrate any significant changes. Low-dose norepinephrine infusion was briefly initiated to maintain systolic blood pressure greater than 140 mmHg for initial kidney reperfusion. Arterial blood gas analysis performed postreperfusion demonstrated a significant base deficit and an increased serum potassium (K) (6.0 mmol/L), which was treated with a bolus of 10 units of insulin and a 30 mL of 50% dextrose. Repeat arterial blood analysis at the end of the case demonstrated a serum K of 5.2 mmol/L (Table 1). The patient was extubated in the operating room and transported to the intensive care unit (ICU) in stable condition. The patient received a total of 3 L Plasmalyte and 1.5 L albumin and had 75 mL of urine output after reperfusion.

On postoperative day (POD) 1, immunosuppression was started using tacrolimus (1 mg twice a day) and mycophenolate mofetil (1000 mg twice a day). On POD 2, the tacrolimus was increased to 2 mg twice a day due to a low-serum concentration, and the mycophenolate mofetil was decreased to 500 mg twice a day due to leukocytopenia. The patient was out of bed and ambulating on POD 2. Pain was managed with scheduled acetaminophen and oxycodone. The patient was discharged from the ICU on POD 4.

## 3. Discussion

We describe a case of uncomplicated LDKT performed 28 years after HT in a patient born with HLHS. HT for HLHS is generally associated with good survival. Our patient was one of the longest survivors described in literature. At the time of KT, she was in relatively good health and had no limitations to her activities of daily living.

The etiology of HLHS is most likely genetic. A mutation in the cardiac transcription cardiac factor NKX2.5 has been demonstrated in patients with HLHS [9, 10]. It has also been shown that a significant percentage of infants with HLHS have first-degree relatives with congenital heart defects [11]. In addition, other genetically related conditions such as Jacobsen syndrome (terminal 11 deletion) have been associated with HLHS [12]. Several environmental (exposure to teratogens), gestational, maternal, and familial factors are also known to predispose patients to develop HLHS [1].

Affected children require HT or surgical palliation in the neonatal and early childhood period with a series of 3 staged procedures: a Norwood procedure in the 1^st^ week of life, Bidirectional Glenn or Hemi-Fontan at 3-6 months of age, and finally, a Fontan procedure at 2-5 years of age [13]. Palliative repair is preferred as infant heart donors are rare. In the past, HT was the only option with acceptable long-term outcomes. Currently, most experts do not feel that HT offers any significant benefit compared to surgical palliation [13].

HT performed in infancy is associated with excellent results. Graft survival (the major limiting factor) at 25 years is about 60% [14]. The reasons for this excellent survival in infants include an immunological advantage (decreased T-cell receptor diversity [15]), decreased waiting time for a HT in comparison to adults, and lack of medical comorbidities at the time of HT [14].

In comparison to HT performed in infancy, adults requiring HT due to adult congenital heart disease (ACHD) have worse outcomes [16]. Adults frequently have longer waiting-list time due to more restrictive selection criteria [17]. The prolonged waiting list time is associated with an increase in heart failure-related complications. It has been demonstrated that the use of either an implantable cardiac defibrillator or ventricular-assisted device did not improve outcomes in patients with ACHD [16, 18]. The diagnosis of ACHD is associated with an additional postoperative mortality of 16%-19% versus 6%-9% after HT for other causes [17, 19]. Mortality after HT in patients with ACHD is related primarily to early hemorrhage and posttransplant cerebrovascular events as well as with the use of high risk donors [19, 20]. Is has also been demonstrated that patients with ACHD underdoing retransplantation have a 2.75-fold increased hazard ratio for dying within 5 years in comparison to patients with ACHD having a primary HT [21].

ESKD in pediatric patients after HT significantly increases mortality [6]. The same has been shown for patients with ACHD [19]. CKD in this population is frequent and related to immunosuppression (calcineurin inhibitors (CNI)) and hypertension [14]. It has also been demonstrated that HT itself, performed before kidney maturity, also contributes to the development of CKD [14].

CNI-induced nephrotoxicity is due to a direct toxic effect on the renal tubule secondary to the high concentration of tacrolimus in tubular epithelial and endothelial cells [22]. Tacrolimus also causes vasospasm which reduces glomerular filtration [23]. Administration of CNIs may also be responsible for posttransplant hypertension due to inhibition of nitric oxide production [24] and increased endothelin release [25].

Azathioprine-related interstitial nephritis usually resolves after azathioprine withdrawal [26]. Its toxicity typically does not cause long-term injury.

A debate continues over the allocation of kidneys for transplantation in a recipient of nonrenal solid organs. It was previously felt that patients receiving a KT after prior organ transplantation may conflict equity standards. This individual would receive an additional donated organ while others are waiting for their first. According to the U.S. Department of Health and Human Services (2019), about 12% of patients waiting for a KT in the United States had a prior organ transplant [27]. Cassuto et al. found that prior nonrenal transplant recipients relisted for other organ transplant had a greater risk of mortality or delisting compared to patients awaiting an isolated KT. Specifically in prior HT recipients, the risk of death was 192% higher [28]. Despite this increased risk, there remains a survival benefit in HT patients receiving a KT [28]. This survival benefit may offer these patients a leg up when being considered for KT compared to primary KT patients.

Our patient had some degree of regurgitation across all four cardiac valves. Tricuspid valve regurgitation (TVR) is one of most frequent valvular complications after HT with an incidence of over 80% [29]. TVR occurs secondary to distortion of the tricuspid annulus at the time of HT in addition to progressive annular dilation over time [30]. Another cause of TVR is a mismatch between donor/recipient pericardial cavity [31]. Allograft rejection and increased pulmonary pressure of recipients are also causes of TVR [31, 32]. Other valvulopathies commonly occurring after HT are pulmonary and mitral regurgitation (PVR and MVR). The incidence of PVR and MVR can be as high as 42% and 32%, respectively, and have a similar pathophysiology to TVR [33]. These valvulopathies require medical management and, if progressive, require surgical correction and possibly retransplantation.

Our patient had sinus bradycardia that required pacemaker placement. Arrhythmias occur frequently after HT and can significantly affect postoperative morbidity and mortality as well as quality of life. Arrhythmias after HT include sinus bradycardia, atrial fibrillation/flutter, ventricular tachycardia, and heart block. The mechanism of arrhythmias is multifactorial and includes conduction system injury during prolonged graft ischemia, surgical technique affecting the sinus node, and sympathetic and parasympathetic denervation with variable degrees of reinnervation over time [34]. Allograft vasculopathy can also lead to arrhythmias necessitating implantation of a cardioverter defibrillator [34]. Bradycardia can be medically managed but often requires placement of a permanent pacemaker in up to 20% of cases [35, 36].

In this report, we describe a case of LDKT performed 28 years after HT for HLHS. Prior to LDKT, the patient enjoyed a good quality of life with excellent functional status. She was continues to be employed as an elementary school teacher. Our patient is one of the longest living HT recipients (surviving without retransplantation) described in the literature. In comparison to adults, HT performed in infancy offers superior long-term survival and excellent quality of life.

## Figures and Tables

**Table 1:** 

	Baseline ABG	Prereperfusion	Postreperfusion	Preextubation
pH	7.452	7.387	7.397	7.368
CO2 (mmHg)	31.2	38.5	37.5	40.9
O2 (mmHg)	83	153	155	119
BE (mmol/L)	-2	-2	-6	-2
HCO3 (mmol/L)	21.9	23	23.3	23.3
Hgb (g/dL)	10.5	10.9	9.5	8.5
HCT (%)	31	32	28	25
Na (mmol/L)	132	129	128	130
K (mmol/L)	4.4	4.8	6.0	5.2
Ca (mmol/L)	0.91	1.01	1.21	1.14

## Data Availability

All data was made available with patients' consent from Penn State Hershey EMR.
